# Pentaploid Wheat Hybrids: Applications, Characterisation, and Challenges

**DOI:** 10.3389/fpls.2017.00358

**Published:** 2017-03-17

**Authors:** Sriram Padmanaban, Peng Zhang, Ray A. Hare, Mark W. Sutherland, Anke Martin

**Affiliations:** ^1^Centre for Crop Health, University of Southern Queensland, ToowoombaQLD, Australia; ^2^Plant Breeding Institute, The University of Sydney, SydneyNSW, Australia

**Keywords:** *in situ* hybridisation, interploidy crosses, pentaploid hybrids, *Triticum aestivum*, *Triticum turgidium* spp. *durum*

## Abstract

Interspecific hybridisation between hexaploid and tetraploid wheat species leads to the development of F_1_ pentaploid hybrids with unique chromosomal constitutions. Pentaploid hybrids derived from bread wheat (*Triticum aestivum* L.) and durum wheat (*Triticum turgidum* spp. *durum* Desf.) crosses can improve the genetic background of either parent by transferring traits of interest. The genetic variability derived from bread and durum wheat and transferred into pentaploid hybrids has the potential to improve disease resistance, abiotic tolerance, and grain quality, and to enhance agronomic characters. Nonetheless, pentaploid wheat hybrids have not been fully exploited in breeding programs aimed at improving crops. There are several potential barriers for efficient pentaploid wheat production, such as low pollen compatibility, poor seed set, failed seedling establishment, and frequent sterility in F_1_ hybrids. However, most of the barriers can be overcome by careful selection of the parental genotypes and by employing the higher ploidy level genotype as the maternal parent. In this review, we summarize the current research on pentaploid wheat hybrids and analyze the advantages and pitfalls of current methods used to assess pentaploid-derived lines. Furthermore, we discuss current and potential applications in commercial breeding programs and future directions for research into pentaploid wheat.

## Introduction

The two major wheat species, hexaploid bread wheat *Triticum aestivum* L. (2n = 6x = 42) and tetraploid durum wheat *T. turgidium* spp. *durum* (2n = 4x = 28), are commercially important wheat species globally. Hexaploid wheat has three diploid sets of seven chromosomes belonging to the A-, B-, and D-genomes (AABBDD), whereas tetraploid wheat only has two diploid sets of seven chromosomes belonging to the A- and B-genomes (AABB). Hybridisation between these two species with different ploidy levels leads to a pentaploid hybrid (AABBD) that has the chromosomal constitution of 2n = 5x = 35 (Kihara, 1924). The genetic variability that is combined from hexaploid and tetraploid wheat into a pentaploid hybrid has great potential in crop improvement (Eberhard et al., 2010; Martin et al., 2013; Kalous et al., 2015). However, while several reviews have focussed on the successful establishment of interspecific wheat hybrids (Sharma and Gill, 1983; Jiang et al., 1993; Friebe et al., 1996), little emphasis has been placed on developing efficient methods to incorporate these pentaploid hybrids into commercial breeding practices.

Although pentaploid wheat hybrids can be efficiently used in crop improvement programs, pest and disease resistance have principally been transferred into hexaploid or tetraploid wheats through alien introgression. Sharma and Gill (1983) reviewed the status of wide hybridisation and listed successful crosses that had been established between wheat and its related genera. They also focussed on the genes that had been successfully transferred from related wild species into commercial wheat cultivars up to that time. Jiang et al. (1993) discussed further advances in successful alien gene transfer from related species into cultivated bread and durum wheat. Friebe et al. (1996) comprehensively reviewed a number of important wheat-alien translocations and their potential in plant breeding for developing pest and disease resistance. Complications occur when trying to introgress traits across different wheat species. These include incompatibility between different *Triticum* species and sterility of the F_1_ hybrids. Developing wheat hybrids through alien introgression is highly challenging when compared to hybridisation between domesticated inter-ploidy species such as bread and durum wheat. Recently, there has been a renewed interest in developing hexaploid/tetraploid wheat crosses to improve elite bread and durum wheat lines for a number of economically desirable characters (Martin et al., 2011, 2013; Han et al., 2014, 2016; Kalous et al., 2015).

This review first focusses on recent research into the production of hexaploid/tetraploid- and tetraploid/hexaploid-derived pentaploid hybrids, mainly between hexaploid *T. aestivum* and tetraploids *T. durum, T. timopheevii*, and *T. dicoccoides*, and then discusses current techniques for characterizing the chromosome composition of lines derived from them. In pentaploid wheat hybrids, the predominance of heterozygous loci present in their A and B genome, together with the retention of a haploid D genome, results in breeding material that has captured a high degree of genetic variation. Despite this rich source of genetic variation, there is still much to learn regarding the efficient screening, selection, and application of populations derived from pentaploid wheat hybrids in commercial breeding programs. This review will examine these challenges and consider the future potential of pentaploid wheat hybrids in crop improvement.

## Genetic Variability in Pentaploid Wheat Hybrids

### Chromosome Morphology

Combining two or more different genomes into one cell may cause changes in chromosome morphology, including differences in the size, thickening, or lengthening of chromosomes, a phenomenon referred to as genome shock (Navashin, 1934). Genome shock has been well-documented (Matsuoka, 2011) and, in addition to changes in morphology, includes chromosomal rearrangements, gain or loss of chromosomal segments, gene activation and suppression, variations in the epigenome especially with respect to the pattern of cytosine methylation, and activation of transposons (Matsuoka, 2011). Increases in genome dosage and genes in alloploid wheat lines causes chromosomal imbalance, genome instability, and incompatibility. For example, morphological changes were observed in the satellite D chromosome in an interploidy cross in *Crepis* species. These observations indicated that there were chromosomes that lacked satellites in the F_1_ progeny (Pikaard, 1999).

### Chromosomal Constitution

**FIGURE 1:**
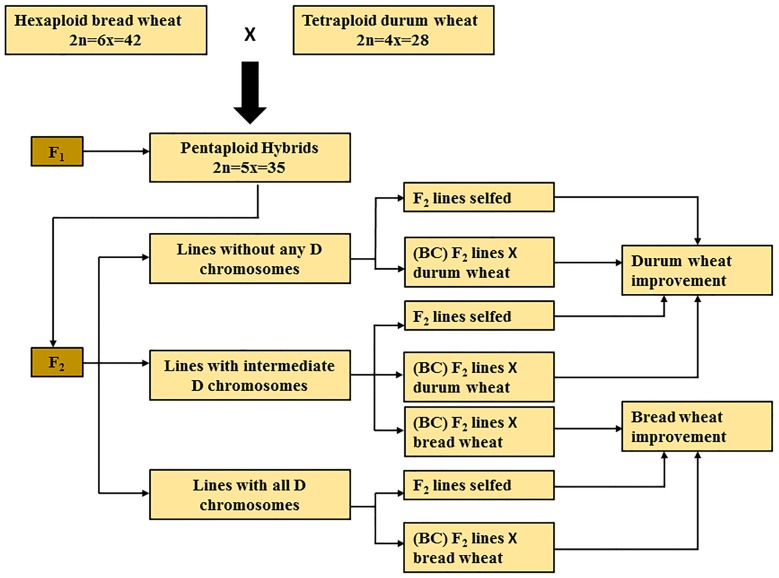
**Summary of pentaploid wheat production for bread and durum wheat improvement.** BC, back crossing.

Chromosome elimination is an essential process that takes place in subsequent generations derived from F_1_ pentaploids, It may take a few generations to resolve the complex process of chromosome pairing and to give rise to a stable durum or bread wheat line. Subsequent generations derived from F_1_ pentaploid wheat hybrids can be broadly classified into three groups, based on the presence or absence of D-genome chromosomes (**Figure 1**). The progeny belonging to the first group have lost all seven D-genome chromosomes (2n = 4x = 28); the second group consists of progeny that have intermediate numbers of D-genome chromosomes (total chromosome numbers ranging from 2n = 29 to 41); while the third group have retained two copies of all seven D-genome chromosomes (2n = 6x = 42). Based on the objective of the breeding program that aims to develop bread or durum wheat lines, these three groups of pentaploid-derived wheat hybrids can by selfed or backcrossed with either parent. For example, lines belonging to the first group of hybrids can be selfed or backcrossed to a durum parent to develop elite durum lines (**Figure 1**).

To determine the general fate of D-genome chromosomes in the lines derived from F_1_ pentaploids, we analyzed previous studies of pentaploid-derived populations from different generations of hexaploid/tetraploid and tetraploid/hexaploid crosses. The number of D-genome chromosomes retained was determined using cytological characterisation (Kihara, 1982; Wang et al., 2005; Eberhard et al., 2010), or molecular markers (Lanning et al., 2008; Eberhard et al., 2010; Martin et al., 2011; Kalous et al., 2015). Molecular marker data indicates the presence or absence of unique chromosomes, but fails to determine the number of copies of unique chromosomes. The cytological studies used in the analysis identified the number of copies of unique chromosomes present, but, did not distinguish individual chromosomes. For this analysis the cytological and molecular data that represent D-genome retention were collected and divided into two main groups. Group-1 includes (i) lines without any D-genome chromosomes and (ii) lines with an intermediate number of D-chromosomes, including lines which had lost both copies of at least one unique D-genome chromosome. With the latter the assumption was made that these lines would loose their D chromosomes in later generations and revert back to a tetraploid constitution. Group-2 includes lines which retained at least one copy of all seven D-genome chromosomes.

**FIGURE 2:**
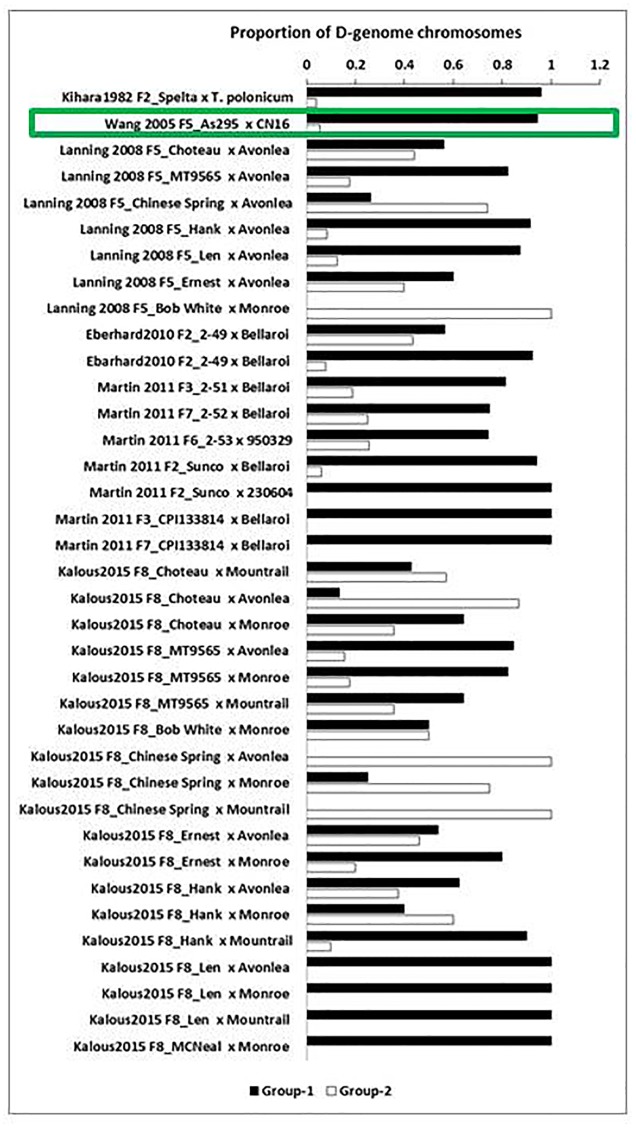
**Summary of D-genome chromosome retention in hexaploid/tetraploid and tetraploid/hexaploid crosses.** Durum wheat Group-1 (lines with no or intermediate number of D chromosomes) is indicated in black and bread wheat Group-2 (lines with at least one copy of all seven D chromosomes) in white. The cross which has a tetraploid maternal parent is indicated with a green box. The proportion of D-genome chromosomes retained in each cross is indicated on the X-axis.

The proportion of D-genome chromosomes retained within each group was calculated for each cross (Supplementary Table 1). The main finding from the D genome retention analysis is that there is a high probability that viable progeny derived from pentaploid wheat hybrids will lose their D-genome chromosomes in subsequent generations (*P* < 0.001), resulting in stable tetraploid lines (Supplementary Table 1). Differences were observed in the retention of D-genome chromosomes depending on the parents used in the cross. For example the hexaploid parent Choteau crossed with tetraploid parents Mountrail and Monroe, resulted in a larger number of D chromosomes being eliminated compared to the F8_Choteau/Avonlea cross (**Figure 2**). In the tetraploid/hexaploid cross involving tetraploid parent AS295 as female parent with hexaploid parents CN16 as male parent, a large proportion of D-genome chromosomes was eliminated (**Figure 2**).

Crosses involving Chinese Spring as hexaploid maternal parent and different tetraploid paternal parents retained high proportions of D-genome chromosomes. These lines are potentially useful for developing bread wheats. Conversely, Martin et al. (2011) observed that crosses involving the hexaploid variety Sunco and tetraploid breeder’s line 230604 and those involving the synthetic hexaploid CPI133814 lost their D-genome chromosomes in early generations. Thus, the parental combinations used in pentaploid crosses greatly influence the degeree to which D-genome chromosomes are retained.

### Relationship between Retention of Tetraploid-Derived A and B Genome and Hexaploid-Derived D Genome

Genomic analysis of generations derived from pentaploid F_1_ lines indicated a significant relationship between the proportion of tetraploid-derived A and B genome retained and the retention of the D genome (Martin et al., 2011). These authors showed that the relative inheritance of chromosomes A and B alleles from bread and durum wheat differed among the hexaploid/tetraploid crosses. Lines with higher levels of durum-derived A and B chromosome segments tended to retain fewer D genome chromosomes. This implies that for characters inherited from the A and/or B genome of the hexaploid parent, pentaploid-derived lines with both low numbers of D chromosomes and the characters in question should be selected. Subsequent backcrossing will more rapidly yield elite tetraploid lines that have both lost all D genome material and inherited the targeted traits of the original hexaploid parent.

## Applications of Pentaploid Wheat Hybrids

Pentaploid wheat hybrids are a potential source for developing resistance to pests and diseases and for improving the level of tolerance to abiotic stresses such as salinity and metal toxicity (Munns et al., 1999; Lopes et al., 2010; Han et al., 2016). Improving quality characteristics is one of the primary concerns when developing commercial varieties suitable for human and animal consumption. For example, grain protein content, protein quality, flour color, and grain texture are key quality criteria when developing wheat varieties for bread and pasta production (Sissons, 2008).

### Disease Resistance

Crown rot, Fusarium head blight (FHB), leaf rust, stripe rust, stem rust, and powdery mildew are among the most devastating diseases of wheat that account for significant yield losses in terms of both quality and quantity (Rong et al., 2000; Bai and Shaner, 2004; Xu et al., 2013; Liu and Ogbonnaya, 2015). Crown rot disease caused by the fungus *Fusarium pseudograminearum* is considered to be one of the major constraints in durum production worldwide (Liu and Ogbonnaya, 2015). This is a chronic and severe disease in many semi-arid regions globally. In the Pacific Northwest of the USA, winter wheat crop yield reduction due to crown rot has been estimated at 35% (Smiley et al., 2005). To date, resistance to crown rot disease in durum and related tetraploid wheat species has not been identified. Partial resistance to crown rot has been identified in some bread wheat lines, such as Sunco, 2-49, and CPI133814 (Wildermuth et al., 2001; Bovill et al., 2006, 2010; Martin et al., 2015). This partial resistance in the hexaploid source is associated with multiple chromosomal regions, including 1A, 1B, 1D, 3B, and 4B in 2-49, and 2B in Sunco (Bovill et al., 2010; Martin et al., 2015). Subsequent hexaploid/tetraploid crosses were made using these partially resistant sources (2-49 and Sunco) with durum breeder’s line 950329. F_6_ lines were selected, based on the complete absence of D-genome chromosomes and crown rot score, and backcrossed with durum parent 950329. The BC_2_F_2_ progeny were assessed for crown rot severity and lines were identified with field-based resistance better than that of 2-49. The results of this study indicate that the hexaploid source of crown rot resistance was successfully introgressed into durum wheat (Martin et al., 2013).

Fusarium head blight is another important wheat disease caused by the fungus *Fusarium graminearum*, which results in a loss of yield and grain quality (Clear and Patrick, 1990). Losses due to reduced yield by FHB have been estimated to be US $2.7 billion (Matny, 2015). The production of mycotoxins by *F. graminearum* in cereal grain, particularly in wheat, is of great concern, rendering the grain unsuitable for human and animal consumption (Bottalico and Perrone, 2002). Chinese bread wheat variety Sumai3 and the two cultivars Ning8331 and 93FHB21 have been identified as resistant sources for FHB. They were crossed with the susceptible durum cultivars Stewart 63 and DT486, and the resulting F_1_ pentaploid hybrids showed improved resistance to FHB compared to the durum parent (Gilbert et al., 2000).

Stripe rust caused by the fungus *Puccinia striiformis* f. sp. *tritici* is an important disease that causes major damage in both durum and bread wheat and can cause severe yield losses of more than 90% in susceptible cultivars when the weather is favorable to disease development (Chen, 2013). The Ethiopian durum wheat line accession PI 480148 has a single dominant gene *Yr53* that confers stripe rust resistance. *Yr53* was transferred into the susceptible bread wheat genotype Avocet S through pentaploid crossing (Xu et al., 2013). The progeny derived from the crosses were cytologically selected based on the presence of all seven pairs of D chromosomes (2n = 6x = 42) and tested with stripe rust race PST100. The progeny of the F_3_ generation segregated in a 3:1 resistant: susceptible ratio, suggesting that a single dominant gene was responsible for the resistance.

Powdery mildew caused by the fungus *Blumeria graminis* f. sp. *tritici* is one of the major diseases that cause significant yield loss in wheat. Pentaploid hybrids derived from crossing susceptible hexaploid wheat cultivars Maris Nimrod and Norman with resistant tetraploid *T. dicoccoides* accession CLI060025 showed improved resistance to powdery mildew when the stable F_3_ progeny were back- and top-crossed to a second hexaploid wheat (Reader and Miller, 1991). In another study, Rong et al. (2000) transferred the powdery mildew resistance gene *Pm26*, present on chromosome 2B from an Israeli *T. dicoccoides* accession (TTD140), into the hexaploid cultivar Bethlehem.

### Abiotic Stress Tolerance

Salt intolerance is a constraint that limits durum wheat production in Australia. Significant variability in saline tolerance exists in the tribe Triticeae, providing a considerable potential for transferring salt-tolerant traits into cultivated bread and durum wheat through pentaploid production (Colmer et al., 2006). Bread wheat is generally more tolerant to salinity than is durum wheat, due to the presence of salt-tolerant genes on the D-genome chromosomes (Munns et al., 1999). The major locus responsible for tolerance to salinity, *Kna1*, has been mapped to the distal end of 4DL (Lindsay et al., 2004). It has been shown that it is possible to improve the saline tolerance in durum wheat by introducing D chromosomes through wheat substitution lines. Langdon durum substitution lines, developed by Joppa and Williams (1988), have a pair of homoeologous chromosomes replaced by a pair of D chromosomes derived from the hexaploid wheat landrace Chinese Spring. These lines have opened up new avenues for developing durum wheat with improved tolerance to various abiotic stresses, including saline tolerance. A Langdon durum 4D (4B) substitution line was crossed with an Australian semi-dwarf durum wheat variety, Jandaroi, to incorporate aluminum tolerance. The chromosomal fragment of 4D was successfully introduced into the Jandaroi durum wheat, which substantially enhanced aluminum tolerance in the sister lines derived from three generations of backcrossing (Han et al., 2014). Two genes, *TaALMT1* and *TaMATE1B*, were transferred using a *Ph1* (pairing homoeologous) mutant of durum wheat through conventional breeding. The size of the 4D chromosomes introgressed from the bread wheat into durum wheat was estimated by markers, fluorescence *in situ* hybridisation (FISH), and real time quantitative PCR. The *TaALMT1* and *TaMATE1B* genes increased Al^3+^ tolerance in durum wheat and, in contrast to bread wheat, the *TaMATE1B* gene was found to be more effective in increasing Al^3+^ tolerance in durum wheat grown on acid soil (Han et al., 2016).

### Quality Improvement

Understanding the molecular, chemical, and functional aspects of the quality of bread and durum wheat has significantly improved in recent decades (Shepherd, 1988; Shewry et al., 1992; Liu et al., 1996; Troccoli et al., 2000; Zhang et al., 2008). Durum wheat is mainly used for the production of pasta, semolina, couscous, and some bread products (Palumbo et al., 2000; Sissons, 2012). Durum has the hardest texture of rich yellow starchy endosperm among all of the wheat species, which makes it the most suitable for pasta production. Protein content and gluten quality are important traits in defining pasta-cooking quality; thus, the quality of the durum grain is directly associated with pasta quality (Novaro et al., 1993).

Flatbread made of durum flour is popular in the Mediterranean region and has also become popular in other countries. The inextensible dough character of durum wheat flour results in a lower loaf volume than bread wheat flour. Introgression of certain traits associated with the dough quality from bread wheat into durum wheat might improve the loaf quality of durum wheat. Palumbo et al. (2000) transferred a high molecular weight gluten portion present on chromosome 1A (*Glu-A1*) that is absent in most of the durum wheat cultivars, through interspecific hybridisation with a bread wheat variety. The resultant interspecific lines showed improved bread loaf volume (cm^3^), ranging from 295 to 442.5, when compared to the tester bread wheat lines, which ranged from 390.0 to 437.5. Interspecific hybridisation involving hexaploid/tetraploid crosses has demonstrated that the resultant progeny have significantly improved grain weight, grain diameter, and grain yield (Kalous et al., 2015). Furthermore, it was suggested that introducing 1D chromosome segments and their associated protein products would improve the bread making quality of durum wheat (Sissons, 2008). Wang et al. (2005) indicated that recombinant allohexaploid lines, which had retained all seven copies of the D genome chromosomes, had enhanced protein content compared to their hexaploid parental lines. However, this study did not indicate which D genome regions are responsible for the improved bread making quality.

Allotetraploid lines derived from crossing of hexaploids/tetraploids that lack D-genome chromosomes showed improvement in storage protein content, indicating that several endosperm-protein genes on chromosomes A and B were activated in the absence of D genome chromosomes (Galili and Feldman, 1984). Furthermore they explained that the suppression of endosperm-protein genes might have occurred soon after the emergence of allohexaploid wheat, around 10,000 years ago, when the D-genome chromosomes were introduced into the tetraploid background. Potentially, these tetraploid specific endosperm-protein genes on chromosomes A and B can be re-activated in hexaploid lines that lose D chromosomes, while combining hexaploid/tetraploid and tetraploid/hexaploid crosses in pentaploid wheat production.

## Techniques for Studying Genome Constitutions of Pentaploid Wheat Hybrids

Characterisation of pentaploid wheat hybrids requires techniques to identify chromosome number (e.g., of univalent D chromosomes), chromosome identity, and changes in chromosomal morphology (deletions or translocations). The following section will discuss the advantages and limitations of techniques currently available for characterizing pentaploid-derived wheat hybrids.

### Cytological Characterisation of Pentaploid Wheat Hybrids

Fluorescence *in situ* hybridisation is a technique that can be used to identify chromosomes, and helps distinguish the 21 pairs of A-, B-, and D-genome chromosomes. The orginal technique involved hybridisation of radioactive-labeled DNA or RNA probes but this was subsequently replaced by fluroscence labeling of the probes (Gall and Pardue, 1969). *In situ* hybridisation enables the identification of deletions, translocations, introgressed chromatin fragments, and translocation breakpoints (Le et al., 1989; Schwarzacher et al., 1989; Friebe et al., 1993). The physical position of known DNA sequences on the chromosomes can be visualized with the help of labeled complementary DNA strands, i.e., probes (Rayburn and Gill, 1986; Zhang et al., 2007).

Multicolor fluorescence *in situ* hybridisation (MCFISH) has been widely used for simultaneous discrimination of different genomes in polyploids, incuding cereals. This technique uses probes with dispersed repetitive DNA sequences that preferentially hybridize to the A- (BAC676D4) and D- (BAC9M13) genome chromosomes, respectively. These FISH probes are labeled with fluorescent tags of different colors and together with counterstaining of the unlabeled B-genome will distinguish the three genomes under a fluorescent microscope (Zhang et al., 2004; Komuro et al., 2013). This method has been used in pentaploid wheat hybrid studies to distinguish the A, B, and D genome chromosomes and to determine the copy number of D-genome chromosomes present in F_2_ plants of hexaploid/tetraploid wheat hybrids (Eberhard et al., 2010). It is also possible to identify the individual chromosomes by using chromosome-specific repetitive probes. For instance, plasmid clone pAs1 can be used to discriminate between the individual D-genome chromosomes based on the signal patterns (Mukai et al., 1993; Koo et al., 2015). To differentiate the chromosomes of different genomes in polyploid individuals, the total genomic DNA of one parent is labeled and used as a probe, while a higher amount of unlabeled genomic DNA of the other parent is used to block the common repetitive sequences between the parents and to increase the specificity of DNA hybridisation. This technique is known as genomic *in situ* hybridisation (GISH) and can be used for studying intergenomic translocations and alien introgressions, and for discriminating genomes in polyploid cereals (Schwarzacher et al., 1989, 1992; Schubert et al., 2001; Silva and Souza, 2013).

Even though cytological techniques have improved over the past decades, there are still some disadvantages in utilizing these approaches for screening a large amount of samples. The genome size, homologous nature of diploid donor species and presence of large numbers of repetitive sequence is still challenging for wheat cytogenetics. Furthermore, cytological approaches are labor intensive, demand a high level of technical skill and require extended periods of time for assessment of multiple progeny. The time and skills required render these approaches unsuitable for use as a high-throughput screening method, as would be required for commercial breeding (Eberhard et al., 2010). However, these methods have an important role in fundamental research and in characterizing the chromosome constitution of elite pentaploid-derived lines.

### Molecular Marker Technology

Unlike cytological techniques, molecular markers are not influenced by the environment or plant growth stages. Different types of molecular markers are available depending on their applications, such as hybridisation-based DNA markers, PCR-based markers, DNA chip and sequence-based markers. Applications of these markers in modern plant breeding efforts have been comprehensively discussed elsewhere (Gupta et al., 2008; Wang S. et al., 2014).

Simple sequence repeat (SSR) or microsatellite markers have been widely used and were considered as the marker of choice for many years by plant breeders analyzing interspecific hexaploid/tetraploid crosses (Lanning et al., 2008). SSR markers are readily reproducible and can be used to distinguish the presence or absence of unique chromosomes. Most SSRs are co-dominant markers and can be used to distinguish genotypes based on the size of alleles and are thus also useful for validating F_1_ hybrids. When co-dominant at a particular locus, SSR markers can also indicate whether one or two copies of a particular chromosome segment are present. Several high-density maps containing SSR markers have been constructed for bread and durum wheat (Roder et al., 1998; Somers et al., 2004; Marone et al., 2012).

In the past decade, a number of other marker platforms have been developed, including mircroarray or gene chips that contain 1000s of unique probes spanning the entire wheat genome (Gupta et al., 2008). For large-scale screening, SSR markers have been largely replaced with single nucleotide polymorphism (SNP) markers covering the entire wheat genome with high density (Wang H. et al., 2014). In addition, the DArT (Diversity Array Technology) markers are based on a microarray platform that hybridizes the sample genome to identify the presence or absence of 1000s of unique fragements covering the whole genome (Jaccoud et al., 2001; Akbari et al., 2006). These DArT markers have been used to study genome inheritance and chromosome structure in pentaploid-derived wheat hybrid crosses (Eberhard et al., 2010; Martin et al., 2011). Recently, the DArT genotyping system has been improved by combining next-generation sequencing (NGS) with existing DArT markers to develop DArTseq^TM^. DArTseq^TM^ markers have the potential to significantly increase the number of markers on each chromosome (Von Mark et al., 2013). This technique has been successfully applied to high-throughput screening of genetically diverse plant materials (Ren et al., 2015).

The majority of existing genotyping systems are based on dominant markers, which fail to differentiate between the presence of a single or two copies of a particular locus. Thus, it is impossible to detect incomplete or partial chromosomes in the presence of a complete homologous chromosome. Hence, the information generated through molecular markers alone is not sufficient to validate the allotetraploid or allohexaploid lines that show chromosome deletions, additions, or translocations. In such instances, it is essential to apply cytological techniques, such as GISH or MCFISH, which provide a more systematic approach for analyzing complex chromosomal complements (Eberhard et al., 2010).

## Challenges of Producing Pentaploid Wheat Hybrids

Pentaploid hybrid production can be complex and requires careful consideration before any crosses are commenced. Common difficulties encountered and important points to consider while developing pentaploid hybrids are summarized below.

### Cross Direction (Hexaploid/Tetraploid or Tetraploid/Hexaploid)

To obtain the highest number of fertile F_1_ progeny from an interspecific cross, it has been proposed that the higher ploidy level species should be used as the maternal parent (Kihara, 1982). In most studies of pentaploid hybrids to date, a hexaploid wheat has been used as the maternal parent (Mesfin et al., 1999; Lanning et al., 2008; Eberhard et al., 2010; Martin et al., 2011; Kalous et al., 2015). Crosses using the lower ploidy level species as the female generally have been less successful and can lead to poor seed set and subsequent low levels of seed germination and seedling establishment (Sharma and Gill, 1983). However, successful pentaploid hybrid crosses combining tetraploid wheat as the maternal parent and hexaploid wheat as the paternal parent have been reported (Wang et al., 2005).

### Seedling Abnormailities

The F_1_ seeds from inter-ploidy hybridisation between bread and durum wheat have relatively poor germination compared to seeds from crosses of parents of the same ploidy level (Kihara, 1982; Sharma and Gill, 1983) and can take several weeks to germinate. Complete failure of germination has been encountered in the reciprocal crosses between *T. monococcum* and *T. aestivum* (Kihara, 1982; Bhagyalakshmi et al., 2008). Unsurprisingly, a strong correlation between seed germination and seed morphology has been observed, with shriveled seeds showing poor germination compared to plump seeds (Kihara, 1982).

In interploidy crosses, normal seed development depends on the ploidy ratio of the maternal and paternal parent (Johnston et al., 1980; Carputo et al., 1999). Each species has been assigned a unique endosperm balance number (EBN). It is generally believed that the ploidy level of an embryo and its associated endosperm is critical for successful seed development (Ramsey and Schemske, 1998). Endosperm development is significant and has a major physiological and genetic relationship with the embryo of the newly formed allopolyploids. Johnston et al. (1980) explained the differences in EBN in an interspecific cross using tetraploid and diploid parent 4EBN(4x)/2EBN(2x) and its reciprocal cross. They indicated that progeny from the 4EBN/2EBN cross had a maternal:paternal ratio of endosperm of 4:1, while the reciprocal cross 2EBN/4EBN had a ratio of 1:1 which deviates from the normal 2:1 maternal:paternal ratio. EBN signifies the importance of endosperm development in inter ploidy crosses.

Even if F_1_ pentaploid seeds germinate normally, chlorophyll abnormalities can develop, such as striato-virescence, delayed virescence, and albino expression (Tsunewaki, 2004). Furthermore, Tsunewaki (2004) showed that chlorophyll abnormalities were observed in the tetraploid wheat species with two duplicated recessive genes controlling the abnormalities. Hexaploid wheat lines carry wild-type homoeoalleles *Sv3*, *Dv3*, and *Abn3* for these abnormalities on chromosome 2D. Furthermore, abnormalities related to growth and development, such as stunted growth, grass clumping, and differences in flowering time, have been reported in interploidy wheat hybrids (Chen and Ni, 2006). While appropriate selection processes can rapidly remove these obvious abnormalities from subsequent pentaploid-derived generations, abnormalities with less obvious phenotypes may be harder to exclude.

### Pollen Viability

Low pollen viability can also restrict seed set. The imbalanced chromosome number in the F_1_ individuals impacts pollen development and subsequent fertilization (Kihara, 1982). The affected pollen grains do not germinate, the pollen tubes fail to reach the ovary, or the male and female gametes fail to fuse (Sharma and Gill, 1983). Pentaploid hybrids derived from a cross between *T. timopheevii* (AAGG) and *T. aestivum* produced completely sterile white anthers with infertile pollen (Bhagyalakshmi et al., 2008). The tissue that forms the pollen grain has a lower threshold for respiratory deficiency than do other plant tissues, which can lead to a loss of pollen viability that has been associated with the expression of novel mitochondrial peptides (Leon et al., 1998). Nucleotide rearrangement in certain genes, including the *atpa* locus, is considered to be the major cause of cytoplasmic male sterility in many crops (Chase, 1994, 2007; Heazlewood et al., 2003).

Apart from pollen viability, several other mutations, such as infertility, leaf striping, and severe growth impairments, could possibly arise from certain combinations of nuclear cytoplasm due to loss of mitochondrial genes (Newton and Coe, 1986). This evidence suggests that the mitochondrial genome may play a vital role in mediating the viability of pollen grains in many species, including interspecific wheat hybrids.

### Progressive Hybrid Necrosis

Progressive hybrid necrosis can affect F_1_ hybrids, resulting in prolonged chlorosis of plant leaf and sheath tissue. These symptoms lead to the premature death of leaves and tillers and eventually the whole plant in certain wheat hybrids (Caldwell and Compton, 1943; Hermsen, 1963a,b). Progressive necrosis is a lethal or semi-lethal condition that imposes a great barrier when trying to transfer desirable traits between species (Chu et al., 2006). This condition in F_1_ hybrids is predominantly controlled by two complementary genes, *Ne1* and *Ne2*, located on the long arm of chromosome 5B and the short arm of chromosome 2B, respectively (Nishikawa et al., 1974). Both genes exist in bread and durum wheats (Tsunewaki, 1970, 1992, 2004). Recent genetic and mutational studies have found that the *Ne2* gene is closely related to the leaf rust resistance gene *Lr13* (Zhang et al., 2016). Knowledge of which bread and durum wheat genotypes carry alleles of the *Ne1* and *Ne2* genes will allow plant breeders to avoid the occurrence of progressive necrosis.

### Nuclear-Cytoplasmic Interaction

Nuclear-cytoplasmic interaction (NCI) is the condition in which an interspecific hybrid possessing the nucleus of one parent interacts with cytoplasm inherited from the other parent (Simons et al., 2003). Pentaploid progeny with an unfavorable NCI exhibit a wide range of phenotypes, such as maternally inherited male sterility, female infertility, late maturity, reduced vigor, pigment deficiencies, and altered morphology of cotyledons, leaves, and flowers (Monika et al., 2013).

The NCI in an interspecific hybrid is expressed by species cytoplasmic specific (*scs*) genes (Monika et al., 2013). In favorable interactions, these *scs* genes maintain the NCI and provide sufficient vigor and viability to the alloplasmic lines (Maan, 1992). The *scs* genes are located on chromosomes 1DL of *T. aestivum* (*scsae*) and 1AL of *T. timopheeviii* (*scsti*). *Triticum turgidum* spp. *durum* has a segment in chromosome 1A carrying *scsti* transferred from *T. timopheevii* (Simons et al., 2003). The *scs* gene plays a major role only when the nucleus of *T. aestivum* or *T. turgidum* spp. *durum* is combined with the cytoplasm of a wild species; otherwise, the *scs* gene stays unexpressed in normal cells with a compatible nucleus and cytoplasm (Monika et al., 2013).

Cytoplasmic organelles such as chloroplast and mitochondria are uniparentally inherited, mainly through the maternal lineage. Inheritance of paternal and novel copies of mitochondrial genes has been witnessed in a number of inter-ploidy wheat crosses. The mitochondrial heteroplasmy in inter-ploidy hybrids between wheat (6x) and rye (2x) (Laser et al., 1997), wheat (6x) and *Aegilops* sp. (2x,4x,6x) (Hattori et al., 2002), wheat (6x) and *T. timopheevi* (4x) (Kitagawa et al., 2003) and between barley (2x) and wheat (6x) (Aksyonova et al., 2005) have all been the subject of study. However, no attention has been given to hexaploid/tetraploid wheat crosses.

Fourty different SNP’s have been identified between the hexaploid bread and tetraploid durum wheat mitochondrial genome. Five of these were present in known mitochondrial genes such as *rps1*, *rps2*, *cox3*, and *ccmFN* (Ogihara et al., 2005; Cui et al., 2009; Noyszewski et al., 2014). Developing SNP markers covering these identified genes would help to differentiate between bread and durum wheat mitochondrial genomes which might provide new insights regarding cytoplasmic inheritance in pentaploid wheat hybrids.

## Conclusion and Future Directions

Although, the value of genetic variability generated from hexaploid/tetraploid or tetraploid/hexaploid crosses is vast this technique has generally not been taken up in plant breeding programs. It is evident from research conducted to date, that pentaploid derived wheat lines would be valuable in commercial plant breeding programs that aim to improve fungal disease resistance, abiotic stress tolerance, quality parameters and agronomic characters. There are a number of other candidate traits, that could be potentially incorporated through pentaploid-derived wheat lines. One of these is nematode tolerance/resistance as durum wheats in general are more resistant to root leison nematode (*Pratylenchus thornei*) disease than bread wheats (Owen et al., 2010; Sheedy et al., 2012, 2015; Thompson et al., 2012). The transfer of some stress tolerant characteristics have been successful, but incorporating drought, cold, and heat tolerance characteristics using pentaploid-dervied wheat lines has not been studied in detail. There is a great potential to improve these traits using interspecific hybridisation as there is a wide variation for stress adaptive traits in the *Triticum* gene pool (Reynolds et al., 2009).

Although there are many barriers that restrict the production of pentaploid wheat hybrids, choice of parental genotype and using the higher ploidy level species as the maternal parent can improve the success rate. Improving the molecular and cytological techniques used to screen recombinant progenies will increase the efficiency of the selection process and help breeders in accelerating pentaploid production. Hence, interploidy hybridisation may be a promising tool for developing wheat genotypes that can cope with changing climate conditions. Therefore, it is essential to initiate further research to incorporate such traits from bread into durum wheat or durum into bread wheat through pentaploid wheat hybrids to assist the sustainable and increased global wheat production which will be required in the future.

## Author Contributions

SP conducted the literature survey and wrote the manuscript. PZ, RH, and MS improved the manuscript by comments and suggestions. AM initiated the project and contributed to manuscript.

## Conflict of Interest Statement

The authors declare that the research was conducted in the absence of any commercial or financial relationships that could be construed as a potential conflict of interest.
